# Exploring Sex Differences in the Neural Correlates of Self-and Other-Referential Gender Stereotyping

**DOI:** 10.3389/fnbeh.2019.00031

**Published:** 2019-02-18

**Authors:** Jonas Hornung, Elke Smith, Jessica Junger, Katharina Pauly, Ute Habel, Birgit Derntl

**Affiliations:** ^1^Department of Psychiatry and Psychotherapy, University of Tübingen, Tübingen, Germany; ^2^Department of Psychiatry, Psychotherapy and Psychosomatics, Medical School, RWTH Aachen University, Aachen, Germany; ^3^JARA-BRAIN Institute Brain Structure-Function Relationships, Forschungszentrum Jülich GmbH and RWTH Aachen University, Aachen, Germany; ^4^LEAD Graduate School and Research Network, University of Tübingen, Tübingen, Germany

**Keywords:** sex differences, self-other appraisal, gender, gender stereotyping, fMRI

## Abstract

While general self-referential processes and their neural underpinnings have been extensively investigated with neuroimaging tools, limited data is available on sex differences regarding self- and other-referential processing. To fill this gap, we measured 17 healthy women and men who performed a self- vs. other-appraisal task during functional magnetic resonance imaging (fMRI) using gender-stereotypical adjectives. During the self-appraisal task, typical male (e.g., “dominant,” “competitive”) and female adjectives (e.g., “communicative,” “sensitive”) were presented and participants were asked whether these adjectives applied to themselves. During the other-appraisal task, a prototypical male (Brad Pitt) and female actor (Julia Roberts) was presented and participants were asked again to judge whether typical male and female adjectives applied to these actors. Regarding self-referential processes, women ascribed significantly more female compared to male traits to themselves. At the same time both women and men indicated a stronger desire to exhibit male over female traits. While fMRI did not detect general sex differences in the self- and other-conditions, some subtle differences were revealed between the sexes: both in right putamen and bilateral amygdala stronger gender-congruent activation was found which was however not associated with behavioral measures like the number of self-ascribed female or male attributes. Furthermore, sex hormone levels showed some associations with brain activation pointing to a different pattern in women and men. Finally, the self- vs. other-condition in general led to stronger activation of the anterior cingulate cortex while the other- vs. self-condition activated the right precuneus more strongly which is in line with previous findings. To conclude, our data lend support for subtle sex differences during processing of stereotypical gender attributes. However, it remains unclear whether such differences have a behavioral relevance. We also point to several limitations of this study including the small sample size and the lack of control for potentially different hormonal states in women.

## Introduction

### Gender Stereotyes

Human beings have self-concepts, i.e., ideas about who they are and expectations about how they should behave in a given situation. These self-concepts also encompass gender stereotypes which are common societal expectations of qualities a woman or man should possess (Cattaneo et al., 2011). The kind of such stereotypes is manifold. Some stereotypes relate to general cognitive skills. For example, the belief that man possess superior mathematical skills seems to exist from early school education onward (Cvencek et al., 2011). The mere existence of such a belief creates a situation of stereotype threat. This term refers the threat of confirming mostly negative stereotypes that exist toward a certain group of individuals and that may impair the functioning of these individuals in a way that confirms the stereotype (Steele and Aronson, 1995).

For example, women show worse mathematical performance when being told that men are superior in a mathemical (Cadinu et al., 2005; Dar-Nimrod and Heine, 2006; Good et al., 2008) or a mental rotation task (Moè and Pazzaglia, 2006; Wraga et al., 2007; Sanchis-Segura et al., 2018). Another kind of stereotype refers to more general psychological qualities. In this regard, women are e.g., more easily associated with low-authority whereas the opposite is true for men (Rudman and Kilianski, 2000; Schmid Mast, 2004). Furthermore, women who do not meet such expectations and appear more agentic, i.e., more independent and competitive, are discriminated against (Rudman and Glick, 2001) and judged as less feminine (Rudman and Glick, 1999). Thus, the existence of such gender stereotypes is likely to have an impact on developmental trajectories of women and men biasing their behavior and attitudes and leading to different educational (Nosek et al., 2009) and occupational outcome (Moss-Racusin et al., 2012). Within the larger framework of gender stereotyping, the present study aimed at exploring how gender stereotypes are represented at the level of brain activation both concerning reflection about oneself and a prototypical woman and man.

### Neural Underpinnings of Self-Referential Processing

Self-appraisal processes and their neurobiological underpinnings have been studied for over a decade. Typically, neuroimaging studies ask adults to respond whether trait words or phrases describe themselves and whether these stimuli can also be attributed to others (for a review see Lieberman, 2007). These self-related processes are especially associated with stronger activation in the medial prefrontal cortex (MPFC) when judging about oneself compared to either close (D’Argembeau et al., 2007; Jenkins et al., 2008; Modinos et al., 2009; Feyers et al., 2010) or famous others (D’Argembeau et al., 2005; Jenkins and Mitchell, 2011). More specifically, studies reported activation of the ventral (van der Meer et al., 2010), dorsal (Fossati et al., 2003), and orbital (Pauly et al., 2013) part of the MPFC depending on the task applied (Northoff et al., 2006). Also, the parahippocampal gyrus and precuneus (Feyers et al., 2010), anterior (Modinos et al., 2009), and posterior cingulate cortex (Johnson et al., 2002) as well as the basal ganglia (Benoit et al., 2010) have been shown to be involved in self-referential processes. Furthermore, Veroude et al. (2013) used an appraisal paradigm, in which participants were instructed to indicate whether a phrase described themselves (self), a friend from college (other), or whether the phrases were positive or negative (control). The authors reported stronger activation in men compared to women in the medial posterior parietal cortex (MPPC) and the bilateral temporo-parietal junction (TPJ) across all appraisal conditions suggesting that sex differences during appraisal of self and others exist. However, up to now it is rather unexplored whether men and women recruit similar or different brain regions during processing of stereotypical female and male attributes.

### Neural Correlates of Other-Referential Processes and Gender Stereotyping

People commonly not only reflect about themselves but also about the characteristics other people possess. Especially the stereotypic gender judgments of others has been shown to recruitthe ventromedial prefrontal cortex (VMPFC), the middle temporal gyrus (MTG), the precuneus and the supramarginal gyrus (Quadflieg et al., 2009). Other studies found an increase in activation with stronger gender stereotyping in the amygdala (Knutson et al., 2007) and a part of the right frontal cortex (Mitchell et al., 2009). However, these studies did not tackle the question whether women and men recruit the same or different brain regions when judging other persons or ascribing gender stereotypical adjectives to them.

### Aims and Expectations

In the present study we aimed at investigating the neural correlates during attribution of gender stereotypes to oneself or to a prototypical female and male actor. The main question of interest was whether participant sex had an impact on stereotype processing while evaluating oneself or a famous other. Specifically, we aimed to explore whether women and men recruit similar brain areas during self- and other-reflection. To our knowledge this question has received almost no attention Veroude et al. (2013) and we therefore refrain from postulating a directional hypothesis regarding such differences. Furthermore, we consider exploratory the impact of female and male sex hormone levels on brain activation during self- and other-reflection. This is because to our knowledge no conclusive model exists regarding the action of sex hormones on human cognition in general let alone on gender stereotyping (Sundström-Poromaa and Gingnell, 2014; Toffoletto et al., 2014).

## Materials and Methods

### Participants

Originally, twenty right-handed healthy Caucasian women and 21 right-handed healthy Caucasian men participated in the study. Participants were recruited via advertisements posted at the RWTH Aachen University, Germany. This study was carried out in accordance with the recommendations of the local Institutional Review Board (EK 088/09) of the Medical School RWTH Aachen University with written informed consent from all subjects. All subjects gave written informed consent in accordance with the Declaration of Helsinki. The protocol was approved by the local Institutional Review Board (EK 088/09) of the Medical School RWTH Aachen University. All subjects were paid for their participation.

The presence of mental disorders was excluded on the basis of the German version of the Structured Clinical Interview for DSM-IV (SCID, Wittchen et al., 1997), which was conducted by experienced psychologists. The usual exclusion criteria for MRI (e.g., metal implants, claustrophobia, and epilepsy) were applied. Independent *t*-tests revealed that women and men were of comparable age (*p* = 0.96) and had similar years of education (*p* = 0.75). Handedness was assessed by means of the Edinburgh Handedness Inventory (Oldfield, 1971) showing that all participants were right-handed apart from one left handed woman and one man. Crystallized verbal intelligence, as assessed with the Mehrfachwahl-Wortschatz-Test Version B (MWT-B, Lehrl, 1996) did not differ between women and men (*p* = 0.67). Moreover, all participants completed the Bem Sex-Role Inventory (Bem, 1981) a standard questionnaire for measuring femininity-masculinity and gender roles.

On the day of testing, a blood sample was taken to assess the sex hormones estradiol, progesterone, and testosterone. Two men did not provide blood samples. Assays were analyzed by the Central Laboratory of the Medical School, RWTH Aachen University, using an electrochemiluminescence-immunoassay (ECLIA, Johnson et al., 1993). The intra-assay accuracy was over 90% (i.e., coefficient of variation was 4–8%) and the sensitivity of each assay was 10 pg/ml (estradiol), 0.2 ng/l (progesterone), and 0.2 ng/ml (testosterone).

#### Exclusion of Participants

Two women and three men were excluded due to faulty logfiles resulting in 18 available behavioral datasets for women and men. Additionally, for fMRI analysis one further woman and man had to be excluded due to strong movements inside the MR scanner (>2 mm in any direction) leading to 17 available datasets for fMRI analysis.

Regarding analysis of hormone levels, extreme values were identified as being larger than 2.5 SDs from the mean of each hormone and separate for each sex. This led to exclusion of two progesterone values for women. In addition to two missing blood samples for men, this resulted in 19 and 20 (men and women) available testosterone values, 19 and 20 (men and women) estradiol values and 19 and 18 (men and women) progesterone values.

Demographic, neuropsychological, and hormonal characteristics of the total sample are shown in Table 1.

**Table 1:** Information on sociodemographic parameters, neuropsychological performance and hormone concentrations in women and men.

	Women *n* = 17	Men *n* = 17	*t*-value	*p*-value
Age (years)	33.71 (13.09)	33.47 (11.29)	0.06	0.96
Education (years)	14.65 (3.39)	14.29 (3.04)	0.32	0.75
Verbal intelligence	112.29 (16.17)	110.18 (12.57)	0.46	0.67
(MWT-B, IQ)
Estradiol (pmol/l)	150.81 (134.97)	89.12 (36.72)*	1.71	0.10
Progesterone (nmol/l)	4.75 (8.88)	2.32 (1.09)*	1.05	0.30
Testosterone (pmol/l)	3.91 (2.20)	33.79 (14.22)*	8.57	<0.001

*Independent t-tests compared women and men. P-values of these tests are indicated. ^∗^Values are given for 15 men as two men did not provide blood samples.*

### Stimuli and Procedure

**FIGURE 1:**
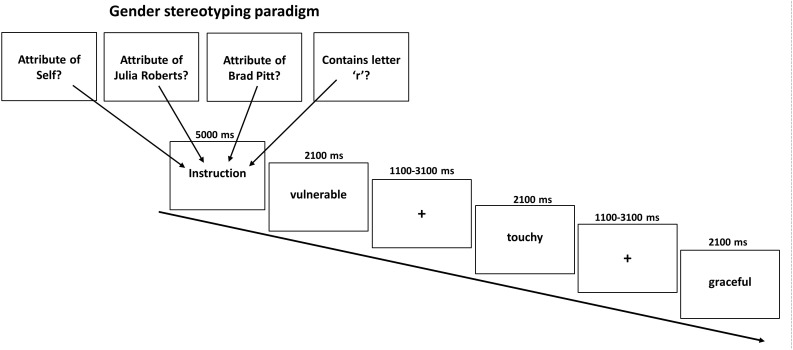
Illustration of the gender stereotyping paradigm. In four conditions typical female or male attributes had to be related to oneself or a typical woman or man. As control a letter judgment condition was used. Each instruction was displayed for 5000 ms before a block of three adjectives was shown (displayed for 2100 ms each) and separated by a jittered fixed cross between 1100 and 3100 ms.

During the task we presented 240 personality traits, half of which had been evaluated as being typical male and the other half as being typical female attributes. The gender typicality of the stimuli was verified in a pre-study in 30 healthy participants (15 women) during which participants rated a total of 240 German adjectives according to whether they were more a prototypical male or female adjective on a continuous scale from -2 (=very masculine) to +2 (=very feminine). Adjectives with an average rating below 0 were labeled as typically masculine whereas adjectives with an average rating above 0 were labeled as typically female. Thus, participants did not necessarily have to agree whether an adjective was more stereotypically female or male which is reflected by a non-zero standard-deviation of ratings. On average, male attributes received a rating of -0.64 (*SD* = 0.33) whereas female attributes were rated on average with 0.72 (*SD* = 0.33). As intended, scores of female and male attributes differed significantly (*p* < 0.001) and to a high degree as indicated by Cohen’s d (*d* = 4.15). All 240 adjectives were used for the main experiment with sixty of these adjectives (balanced for femininity/masculinity) were presented during the self-condition where participants were asked to judge whether the traits applied to themselves or not via button press (left = yes; right = no). Another 120 gender adjectives (60 in each condition) had to be assigned to either a typical male (Brad Pitt) or typical female (Julia Roberts) celebrity. Both had been selected to represent a stereotypical known prototype of a man and a woman. Indeed all participants reported to know who both actors were. In the other-appraisal task participants had to indicate whether the female or male attribute fitted the famous person or not. In a final lexical control condition further 60 female and male adjectives were presented and participants were asked whether the displayed words contained the letter “r” or not. Consequently, the task consisted of 8 experimental conditions in a 2 × 4 event-related design with the factors Attribute (male, female) and Appraisal Condition (self, typical other man, typical other woman, and lexical). This resulted in the following eight conditions (1) self male attributes, (2) self female attributes, (3) prototypical man male attributes, (4) prototypical man female attributes, (5) prototypical woman male attributes, (6) prototypical woman female attributes, (7) lexical male attributes, (8) lexical female attributes. Each condition was presented ten times in mini-blocks of three attributes with the same female and male attributes presented in each condition across participants. As intended, conditions did not differ regarding the mean rating for femininity/masculinity of attributes (*p* = 0.76). A total of 240 stimuli were presented in a pseudo-randomized order. Stimulus presentation was accomplished with Presentation software (Version 14.2, http://www.neurobs.com), whereby each condition was announced by a brief instruction (5 s). Attributes were presented for 2.1 s followed by a fixation cross jittered between 1.1 and 3.1 s. Each last word of a mini-block was followed by a fixation cross jittered between 5.6 and 10.6 s. This resulted in a total task length of about 27 min with no breaks in between (see Figure 1). The order of conditions was permutated to achieve that each condition preceded and followed every other condition approximately equally often to avoid any systematic effects of the order of presentation.

### Analysis of the Behavioral Data

Statistical testing was performed with the Statistical Package for the Social Sciences (SPSS 24, IBM Corp., Armonk, NY, United States). For all analyses, the significance level was set to *p* = 0.05.

Independent *t*-tests were used to compare sociodemographic, hormonal, neuropsychological and questionnaire data between women and men. For BSRI, masculinity and femininity scores were calculated. Additionally, as the BSRI also assesses desired femininity and masculinity, we also compared these scores between women and men via independent *t*-tests.

#### Number of Accepted Female and Male Attributes

Analyses were separated for the self- and other condition. Only adjectives that were agreed on (“yes” answers) were included as due to the dichotomic character of the possible answers, the additional use of “no” answers would result in no further information. Then the number of yes-answers was subject to a 2 × 2 ANOVA with the factor Participant Sex (women, men) and Attribute (female, male) for the self-condition and a 2 × 2 × 2 ANOVA with the additional factor Actor Sex (prototypical male, prototypical female) for the other condition. Greenhouse-Geisser corrected *p*-values are reported in cases of sphericity violation and partial eta squares (η^2^) are listed as an indication of effect size.

##### Behavioral correlation analyses

Behavioral correlation analyses were separately performed for women and men between sex hormones (estradiol, progesterone, testosterone) and behavioral performance (number of self-attributed male, female adjectives).

#### fMRI Data Acquisition and Pre-processing

Functional imaging data were obtained on a 3 Tesla Siemens MR Scanner (Siemens Medical Systems, Erlangen, Germany) at the Department of Psychiatry, Psychotherapy and Psychosomatics of the RWTH Aachen University. Echo-planar imaging (EPI) was applied (T2^∗^, voxel size: 3.1 mm × 3.1 mm × 3.1 mm, distance factor 15%, GAP 0.5 mm, 64 × 64 matrix, FoV: 200 mm × 200 mm, TR = 2 s, TE = 30 ms, α = 76°). Thereby 36 slices in ascending order covering the whole brain were acquired. Image acquisition was preceded by 5 dummy scans, which were discarded before preprocessing. The resulting 815 volumes per subject were analyzed using SPM12 (Statistical Parametric Mapping; Wellcome Trust Centre for Neuroimaging, London, United Kingdom^1^). For preprocessing, functional images were first slice-time corrected, realigned to the first functional image, coregistered with the acquired anatomical image, spatially normalized to the standard template of the Montreal Neurological Institute (MNI, Canada) and finally smoothed with an 8 mm FWHM isotropic Gaussian kernel. To remove effects of low frequency noise, a 128 s high pass filter was used.

### Analysis of the fMRI Data

#### Whole Brain Analyses

On the first level, regressors were modeled for each of the eight experimental conditions and for each subject and subsequently entered into a second level analysis. Here, a flexible factorial design was calculated for the group analyses performing a generalized linear mixed model (GLM). Movement parameters were included as nuisance covariates. Based on this model, (1) main effects of Participant Sex (women, men), Appraisal Condition (self, prototypical male, prototypical female other, lexical), Attribute (male, female) and Acceptance (yes, no) were analyzed. (2) interactions of these factors were also modeled to investigate especially whether sex differences existed during the attribution of male and female attributes (interaction Attribute × Participant Sex) separately in the self- and other conditions. (3) sex differences in the comparison between self- and other-conditions were investigated by modeling the interaction Condition × Participant Sex comparing both other-conditions separately to the self-condition. To adjust for the inflation of α-errors, whole brain analyses were thresholded at *p* < 0.001 (cluster-forming threshold) and family-wise-error corrected (FWE) for multiple comparisons at the cluster level to a threshold of *p* = 0.05. Thus, only clusters with a minimal extent of 70 voxels were detected as significant. The resulting voxel coordinates of significant activation peaks (in MNI-space) were located anatomically by help of an anatomy toolbox (Eickhoff et al., 2005) implemented in SPM12.

#### Regression Analyses

To detect clusters on the whole brain level that significantly covaried with (1) sex hormone levels (testosterone, estradiol, progesterone) and (2) with the ratio of the number of self-ascribed female to male attributes, separate whole brain regression analyses were conducted for women and men. Specifically, the contrast images during the self-condition for female and male attributes from the first level analysis of each participant was covaried with sex hormone levels and the ratio of the number of self-ascribed female to male attributes. To do so, we divided the number of self-ascribed female by the number of self-ascribed male attributes, thus indicating a stronger agreement toward female adjectives with scores larger 1, stronger agreement toward male adjectives with scores smaller 1 and equal agreement between female and male attributes for a score of 1. Again, a cluster-forming threshold of *p* < 0.001 and a FWE-correction at cluster level to a threshold of *p* = 0.05 was performed. Thus, only clusters with a minimal extent of 45 voxels were detected as significant.

#### ROI Analyses

Based on previous studies investigating stereotypical or self- vs. other-processing (Quadflieg et al., 2009; Veroude et al., 2013), we performed several region of interest analyses. These regions included the MPFC, precuneus and bilateral amygdala (Quadflieg et al., 2009) as well as the bilateral TPJ and the MPPC (Veroude et al., 2013). ROIs were defined as 10 mm spheres around center coordinates (in MNI space) taken from these two publications. Only bilateral amygdala was defined anatomically by help of an anatomy toolbox (Eickhoff et al., 2005) to allow for better spatial definition of these ROIs. Mean parameter estimates were extracted and subject to a mixed model 2 × 3 × 2 ANOVA with the factors Participant Sex (men, women), Appraisal Condition (self, prototypical male, female), and Attribute (male, female). Within each ROI, *post hoc* comparisons were Bonferroni-corrected for multiple comparisons. See Table 2 for all ROIs and their spatial extent.

**Table 2:** All brain regions selected for ROI analyses.

Region of				Volume
interest	*X*	*Y*	*Z*	in mm^3^	ROI-definition
L Amygdala	-23	-4	-22	2745	Anatomical
R Amygdala	24	-2	-22	2432	Anatomical
MPFC	-4	54	6	4120	10 mm sphere
Precuneus	10	-58	56	4120	10 mm sphere
Left TPJ	-46	-60	27	3984	10 mm sphere
Right TPJ	49	-63	27	4416	10 mm sphere
MPPC	-4	-56	33	3984	10 mm sphere
R Putamen	27	-18	-7	4792	Derived by functional
					activation

*Center coordinates reflect MNI-space. MPFC, medial prefrontal cortex; MPPC, medial posterior parietal cortex, TPJ, temporo-parietal junction.*

##### Neural correlation analyses

Neural correlation analyses were separately performed for women and men between sex hormones (estradiol, progesterone, testosterone) and the beta estimates during self-processing of female and male attributes in all ROIs.

## Results

### Bem Sex-Role Inventory (BSRI)

Comparing masculinity and femininity scores of women and men via *t*-tests revealed that men described themselves as more masculine (*p* < 0.001) while no significant sex difference emerged for femininity (*p* = 0.88). For the desired self, we did not observe significant sex differences (*t*s < 0.67, *p*s > 0.52). Within-group analyses revealed that both sexes expressed a desire to reveal more masculine compared to feminine traits (both *p*s < 0.001). See Table 3 for statistics.

**Table 3:** Mean scores for self-attributed and desired masculinity and feminity according to the BSRI with the standard deviation in brackets.

	Women	Men	*t*-value	*p*-value
Self male	4.49 (0.49)	5.11 (0.46)	4.02	**<0**.**001**
Self female	4.72 (0.62)	4.68 (0.61)	0.14	0.88
Desired male	2.40 (0.22)	2.35 (0.26)	0.67	0.52
Desired female	2.03 (0.19)	2.01 (0.08)	0.46	0.65

*T- and p-values refer to sex differences. BSRI, bem sex-role inventory.*

### Behavioral Performance

#### Self-Condition

**FIGURE 2:**
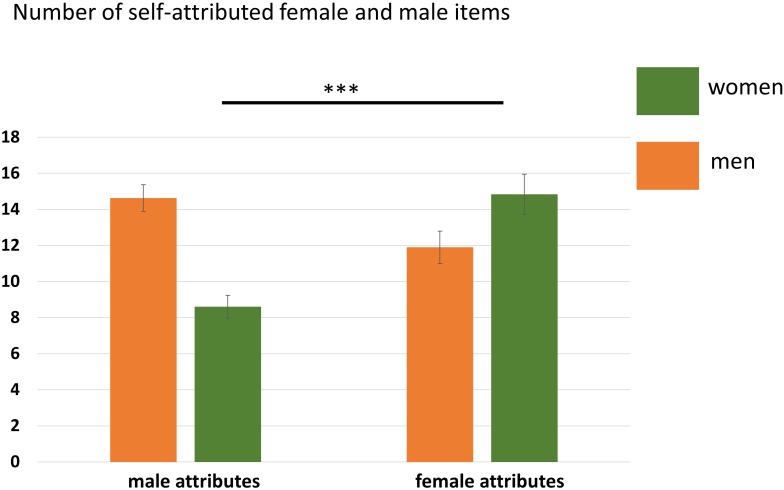
Number of agreed items in the self-condition separated for women and men. Women agreed to significantly more female than male attributes. Error bars indicate the standard error of the mean (SEM). ^∗∗∗^*p* < 0.001.

Neither the main effect of Participant Sex, *F*(1,35) = 3.94, *p* = 0.06, nor the main effect of Attribute, *F*(1,35) = 3.55, *p* = 0.07, reached significance. Only the interaction Participant Sex × Attribute was significant, *F*(1,35) = 23.46, *p* < 0.001, η^2^ = 0.40 indicating that women and men agreed to significantly more gender-congruent items than gender-incongruent items. See also Figure 2 and Table 4.

**Table 4:** Mean number of assigned attributes across the self- and other-conditions (yes-answers) with the standard deviation in brackets.

	Women	Men	*t*-value	*p*-value
Self male	8.61 (2.66)	14.63 (3.14)	6.27	**<0**.**001**
Self female	14.83 (4.77)	11.89 (3.78)	2.08	0.045
Other prototypcial male (male attribute)	16.78 (3.84)	18.47 (3.98)	1.32	0.20
Other prototypical male (female attribute)	14.44 (4.31)	14.68 (4.21)	0.17	0.86
Other prototypical female (male attribute)	12.78 (3.17)	12.52 (3.53)	0.23	0.82
Other prototypical female (female attribute)	18.94 (2.82)	17.47 (3.06)	1.52	0.14

*T- and p-values refer to sex differences.*

#### Other Condition

**FIGURE 3:**
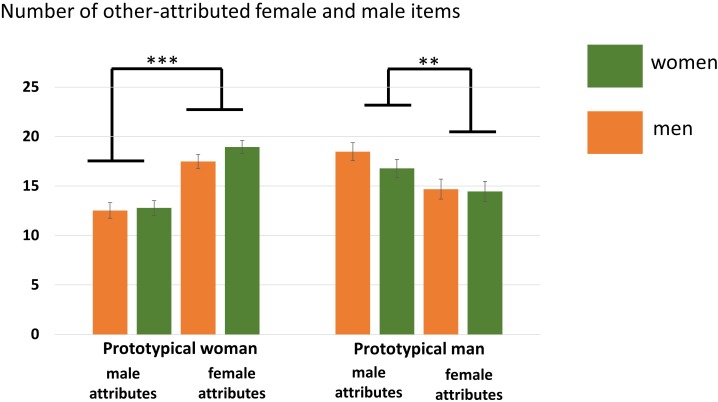
Number of agreed items in the other conditions separated for women and men. More female compared to male attributes were assigned to the prototypical female actor (Julia) while more male compared to female attributes were assigned to the prototypical male actor (Brad). Error bars indicate the standard error of the mean (SEM). ^∗∗∗^*p* < 0.001, ^∗∗^*p* < 0.01.

A main effect of Attribute was found, *F*(1,35) = 7.10, *p* = 0.01, η^2^ = 0.17, showing that overall more male attributes were accepted. Furthermore, an interaction Participant Sex × Actor Sex, *F*(1,35) = 6.99, *p* = 0.01, η^2^ = 0.11, and an interaction Actor Sex × Attribute, *F*(1,35) = 25.07, *p* < 0.001, η^2^ = 0.42, was found. The Participant Sex × Actor Sex interaction indicates that women accepted overall more adjectives for the prototypical female compared to male actor (*p* = 0.02) while this was not the case for men (*p* = 0.34). The Actor Sex × Attribute interaction shows that gender attributes were assigned in an actor-specific manner with more male compared to female attributes (*p* = 0.009) being attributed to the prototypical male actor and more female compared to male attributes (*p* < 0.001) being attributed to the prototypical female actor. No main effect of Actor Sex, *F*(1,35) = 3.69, *p* = 0.06, η^2^ = 0.10, was detected. See also Figure 3 and Table 4.

### fMRI Results

#### Whole Brain Analyses

##### Main effects of self- and other-condition

**FIGURE 4:**
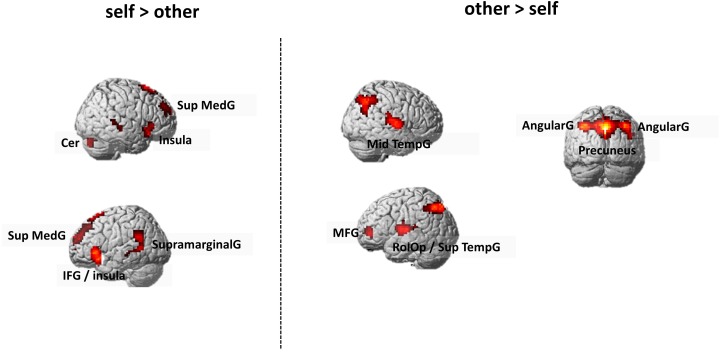
Whole Brain activation in the contrast self > other (left side) and other > self (right side). FWE-correction (*p* < 0.05). AngularG, angular gyrus; Cer, cerebellum, IFG, inferior frontal gyrus; MFG, middle frontal gyrus; Mid TempG, middle temporal gyrus; RolOp, rolandic operculum; SupramarginalG, supramarginal gyrus; Sup MedG, superior medial gyrus; Sup TempG, superior temporal gyrus.

Across all participants, the self-condition (self) compared to the letter judgment condition (lexical) led to strong activation in the left superior frontal gyrus and several smaller clusters including the right temporal gyrus and bilateral cerebellum (see Table 5). The other-condition (other) compared to lexical also led to stronger activation in the left superior frontal gyrus and also involved regions like left inferior frontal gyrus and posterior cingulate cortex (see Table 5). Directly comparing self and other showed stronger activation during self including parts of the left anterior cingulate cortex and supramarginal gyrus. The inverse contrast (other > self) detected stronger activation in the right precuneus and bilateral superior temporal gyrus (see Figure 4 and Table 5).

**Table 5:** Activated brain regions as revealed by whole brain analyses.

Cluster/macroanatomical structure	*x*	*y*	*z*	*t*-score
Self > Other				
Cluster 1 (*k* = 3468 voxels) L ACC	-3	38	5	13.71
Cluster 2 (*k* = 226 voxels) L Supramarginal Gyrus	-47	-49	26	5.16
Cluster 3 (*k* = 134 voxels) R Cerebellum	28	-81	-34	6.09
Cluster 4 (*k* = 78 voxels) R Middle Temporal Gyrus	47	-34	1	4.45
Other > Self				
Cluster 1 (*k* = 1660 voxels) R Precuneus	6	-65	37	9.69
Cluster 2 (*k* = 230 voxels) R Superior Temporal Gyrus	56	-9	1	5.30
Cluster 3 (*k* = 188 voxels) L Superior Temporal Gyrus	-53	-9	5	5.14
Cluster 4 (*k* = 79 voxels) R Posterior-Medial Frontal	3	-21	66	3.68
Cluster 5 (*k* = 70 voxels) L Middle Orbital Gyrus	-41	51	-2	5.64
Self > Lexical				
Cluster 1 (*k* = 5312 voxels) L Superior Frontal Gyrus	-10	54	34	17.81
Cluster 2 (*k* = 224 voxels) R Cerebellum	28	-81	-34	16.48
Cluster 3 (*k* = 222 voxels) R Middle Temporal Gyrus	59	-6	-20	9.09
Cluster 4 (*k* = 71 voxels) L Cerebellum	-28	-81	-34	8.57
Lexical > Self				
Cluster 1 (*k* = 10382 voxels) L Inferior Parietal Lobule	-38	-43	41	15.63
Cluster 2 (*k* = 345 voxels) L Middle Frontal Gyrus	-44	38	26	10.09
Cluster 3 (*k* = 194 voxels) L Cerebellum	-10	-74	-38	6.84
Other > Lexical				
Cluster 1 (*k* = 1552 voxels) L Superior Frontal Gyrus	-10	54	34	16.87
Cluster 2 (*k* = 716 voxels) L IFG	-44	29	-9	14.48
Cluster 3 (*k* = 551 voxels) L PCC	-3	-53	26	15.43
Cluster 4 (*k* = 277 voxels) L Angular Gyrus	-47	-68	30	11.12
Cluster 5 (*k* = 178 voxels) R Cerebellum	28	-81	-34	13.39
Cluster 6 (*k* = 105 voxels) L Hippocampus	-22	-15	-13	5.31
Cluster 7 (*k* = 98 voxels) R Angular Gyrus	53	-65	30	8.50
Cluster 8 (*k* = 91 voxels) R Middle Temporal Gyrus	59	-6	-16	11.74
Cluster 9 (*k* = 85 voxels) R Hippocampus	19	-9	-16	4.45
Lexical > Other				
Cluster 1 (*k* = 9444 voxels) L Inferior Parietal Lobule	-38	-43	44	14.87
Cluster 2 (*k* = 312 voxels) L Middle Frontal Gyrus	-41	41	26	9.82
Cluster 3 (*k* = 241 voxels) L Cerebellum	-10	-74	-34	6.52

*Comparisons of self, other and lexical condition. Coordinates reflect MNI space. ACC, anterior cingulate cortex; IFG, inferior frontal gyrus; *k*, cluster extent; L, left; PCC, posterior cingulate cortex; R, right.*

##### Sex differences during the self-condition

The general effect of Participant Sex pertaining to the contrast women vs. men did not yield any significant clusters. However, the interaction of Attribute × Participant Sex resulted in one significant cluster located in the right putamen (*k* = 137; MNI: *x* = 31 *y* = -6 *z* = -6). To analyse this interaction in more detail, we performed an additional region of interest analysis (see “*A Posteriori* Region of Interest Analysis”).

##### Sex differences during the other-condition

Again, no general Sex effect was detected comparing women vs. men. Also the interaction Attribute × Participant Sex did not lead to significantly activated clusters. Therefore no further *post hoc t*-tests were performed.

##### Sex differences comparing the self- to the other-conditon

Finally, self- and other-conditions were compared by modeling the interaction Condition × Sex for both female and male actor separately. In neither case were significant clusters detected pointing to no differential activation between women and men when comparing self- to other-processing of a typical woman or man.

#### Whole Brain Regression

##### Sex hormones on whole brain activation

###### Women

During presentation of female adjectives no correlation with either hormone was detected whereas during presentation of male adjectives significant correlations were found for both progesterone and testosterone but not estradiol. For progesterone, a cluster (*k* = 90) including left insula and superior temporal gyrus was positively associated with hormone values while for testosterone, a cluster (*k* = 70) in the left postcentral gyrus extending to the rolandic operculum was positively associated with hormone values (see Table 6).

**Table 6:** Resuls of whole brain regression of sex hormone values on brain activation.

	Women	Men
Estradiol	n.s.	R angular gyrus; *k* = 49 (MNI: 54, -52, 30)^∗∗^
Progesterone	L Insula, *k* = 90 (MNI -35, -15, 12)^∗^	R Superior Frontal Gyrus; *k* = 46 (MNI: 25, 7, 55)^∗^
Testosterone	L Postcentral gyrus, *k* = 70 (MNI: -53, -12, 30)^∗^	n.s.

*^∗^Association with male attributes. ^∗∗^Association with female attributes.*

###### Men

During presentation of female adjectives a cluster (*k* = 49) in right angular gyrus was positively associated with estradiol values. Furthermore, during presentation of male adjectives a negative association was found with progesterone values in the superior medial gyrus (*k* = 47). No further significant correlation emerged (see Table 6).

###### Self-ascribed female-to-male-ratio on whole brain activation

Neither in women nor in men the ratio of the number of self-ascribed female to male adjectives was significantly related to whole brain activation.

#### *A Posteriori* Region of Interest Analysis

**FIGURE 5:**
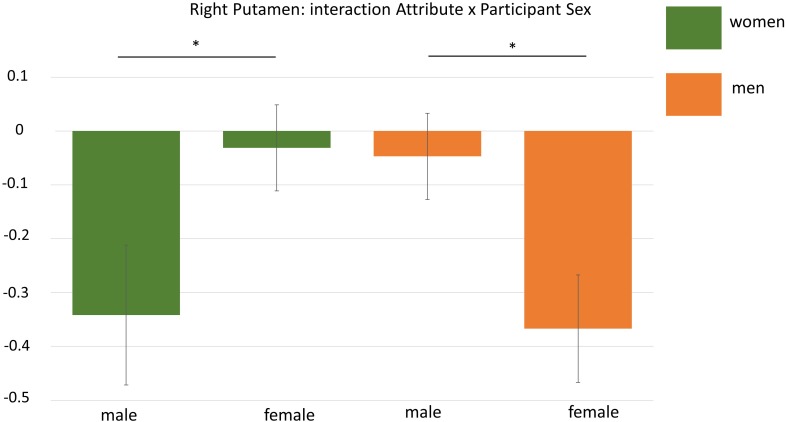
Display of the interaction Attribute × Participant Sex during ROI-analysis in the right putamen. Men showed higher activation for male attributes whereas women had higher activation for female adjectives. ^∗^*p* < 0.05.

As the whole brain interaction Attribute × Participant Sex during the self processing yielded one significant cluster in the right putamen, we extracted mean beta estimates from this cluster for a more detailed analysis. This analysis revealed that not only the interaction Attribute × Participant Sex was significant, *F*(1,32) = 8.66, *p* = 0.006, but that this interaction was additionally dependent on the experimental condition as indicated by a significant three-way interaction Attribute × Participant Sex × Condition, *F*(2,64) = 6.01, *p* = 0.004, η^2^ = 16. To disentangle this three-way interaction, we first computed separate interactions of Attribute × Sex for each condition showing that only for the self-condition this interaction was significant, *F*(1,32) = 28.89, *p* < 0.001, η^2^ = 0.47, but not for the two other conditions (*F*s < 0.17, *p*s > 0.69). This indicates that during self-processing women had higher activation in the right putamen for female compared to male attributes (*p* = 0.001) whereas men showed stronger activation for male compared to female attributes (*p* = 0.002). See also Figure 5.

#### *A Priori* Region of Interest Analyses

##### Main effects of participant sex

Only in the MPFC a main effect of Participant Sex was detected pointing to higher overall activation in men compared to women (*p* = 0.05).

##### Interactions with the factor participant sex

An interaction Attribute × Participant Sex was detected in both left and right amygdala indicating that across all conditions men had by trend a lower activation for female compared to male attributes (left *p* = 0.06; right *p* = 0.08) whereas women did not differ for female and male attributes (left *p* = 0.35, right *p* = 0.32). No further interactions including the factor Participant Sex was detected (*p*s > 0.11, *F*s < 2.27).

##### Main effects of condition and attribute

Please refer to the Supplementary Material and Table 7 for reports of the main effects of the factors Condition and Attribute.

**Table 7:** Region of interest analysis with indication of main effects and interactions with the factor participant sex.

ROI	Factor condition	Factor attribute	Factor participant sex	Sex interactions
L Amy	n.s.	n.s.	n.s.	^∗∗^*F*(1,32) = 4.62, *p* = 0.039, η^2^ = 13
R Amy	n.s.	n.s.	n.s.	^∗∗^*F*(1,32) = 4.69, *p* = 0.038, η^2^ = 13
Precuneus	*F*(2,64) = 5.25, *p* = 0.008, η^2^ = 0.14	n.s.	n.s.	n.s.
L TPJ	*F*(2,64) = 20.26, *p* < 0.001, η^2^ = 0.39	*F*(1,32) = 8.03, *p* = 0.008, η^2^ = 0.20	n.s.	n.s.
R TPJ	*F*(2,64) = 14.06, *p* < 0.001, η^2^ = 0.31	n.s.	n.s.	n.s.
MPPC	*F*(2,64) = 21.04, *p* < 0.001, η^2^ = 0.40	*F*(1,32) = 8.11, *p* = 0.008, η^2^ = 0.20	n.s.	n.s.
MPFC	*F*(2,64) = 74.11, *p* < 0.001, η^2^ = 0.70	*F*(1,32) = 5.27, *p* = 0.028, η^2^ = 0.14	*F*(1,32) = 4.15, *p* = 0.05, η^2^ = 0.12	n.s.
R Putamen	*F*(2,64) = 3.57, *p* = 0.034, eta = 0.10	n.s.	n.s.	^∗^*F*(2,64) = 6.01, *p* = 0.004, η^2^ = 16

*Amy, amygdala; L, left; MPFC, medial prefrontal cortex; MPPC, medial posterior parietal cortex; R, right; TPJ, tempo-parietal junction. ^∗∗^Condition × Participant Sex × Attribute; ^∗^Attribute × Participant Sex.*

#### Correlation Analyses

##### Sex hormones × behavioral data

Sex hormones were correlated separately for women and men with the number of self-ascribed female / male adjectives However, neither in women nor in men, significant correlations were detected data (*r*s < 0.37, *p*s > 0.14).

##### Sex hormones × neural data

For progesterone, in men, a positive association was found with the right (*r* = 0.64, *p* = 0.006) and left (*r* = 0.49, *p* = 0.047) amygdala and left TPJ activation (*r* = 0.52, *p* = 0.32) during presentation of male attributes. All other ROIs were not significantly linked to progesterone values in men and women (*r*s < 0.48; *p*s > 0.05). For estradiol (*r*s < 0.33, *p*s > 0.20) and testosterone (*r*s < 0.33, *p*s > 0.21) no significant correlations were found in men and women. Of note, significant correlations are uncorrected for multiple comparisons as the mere number of comparisons (each hormone was compared separately in women and men with female and male attributes in eight ROIs resulting in 16 comparisons for each sex) would have required almost perfect correlations. We still report these values asking for caution in interpreting them.

## Discussion

The current fMRI study investigated gender-related self- and other-appraisals in adult women and men. The main focus of this study was to investigate whether sex differences on a behavioral and neuronal level exist during such processes.

Notably, women and men self-ascribed more gender-stereotyped traits, i.e., women agreed to have more stereotypical female attributes. At the same time both women and men reported the desire to exhibit more masculine traits. On the level of brain activation, women and men recruited similar brain regions during self- and other-appraisal. Only during self-referential processes one significant cluster in the right putamen was more strongly active pointing to higher gender-congruent activation in women and men, respectively. Furthermore specific region of interest analyses also revealed a similar pattern of gender-congruent activation in bilateral amygdala showing that men had stronger activation for male compared to female attributes – however both during the self- and other-conditions. All other region of interest analyses did not reveal sex differences. Finally, whole brain regressions with sex hormone levels were conducted separately for women and men for the self-condition. The outcome of these analyses yielded different brain regions for women and men including clusters in the left insula and rolandic operculum, right angular gyrus and superior medial gyrus.

### Self-Appraisal of Gender Stereotypes

In our study, we confronted women and men with traits that had been rated as typically female or male in a pre-study. As expected, women and men self-ascribed more gender-congruent attributes. However, only in women this difference reached significance, i.e., women agreed more often to female rather than male traits when referring to themselves. At the same time both women and men reported the desire to exhibit more masculine attributes. A tentative explanation for this observed pattern may consider the occupationaI situation for women who are underrepresented in academic leadership positions and earn less than men in most Western societies (Carnes et al., 2015; Salinas and Bagni, 2017). Such a discrepancy between the sexes seems to be in part due to conscious or unconscious discrimination against women already at the level of applications. For example, data from Moss-Racusin et al. (2012) demonstrate that for identical applications of a bogus female and male student for a position as laboratory managers, men were rated higher on competency and were rather hired and mentored by female and male faculty members. Similary, Steinpreis et al. (1999) found that for identical applications of female and male scientists both female and male reviewers were more likely to hire the male applicants. Interestingly, not only women but also men are discriminated against when applying for jobs that appear not suitable for them (Davison and Burke, 2000) like communal roles including working as a nurse or social worker (Croft et al., 2015). Also in politics more masculine traits appear beneficial for election success. For example, studies by Klofstad et al. (2012) and Anderson and Klofstad (2012) showed that participants listening to differently pitched female and male voices, voted more often for persons with deeper more masculine voices which was true both for female and male candidates. Thus, at least in the above mentioned domains it can be beneficial to exhibit masculine attributes to increase success and therefore the observed desire to exhibit more masculine traits could make sense. However, we want to point out that this narrative is only speculative and cannot explain why male attributes should be preferred in other non-professional contexts. We therefore ask further studies to conduct more detailed and domain-specific investigations to back or refute our speculations about the desire to exhibit more masculine traits which we found for both women and men.

### Sex Differences in Neural Networks of Self- and Other-Appraisal

Previous studies have pointed to differences during the appraisal of self- and other-related attributes with parts of the MPFC being more active during self-referential processing (D’Argembeau et al., 2007; Pfeifer et al., 2007; Veroude et al., 2013). In contrast to this, the precuneus has been most consistently recruited during the retrieval of other-related information (Pfeifer et al., 2007; Quadflieg et al., 2009). More tentative had been results about sex differences during such self- and other-processing. In this regard Veroude et al. (2013) pointed to higher activation of bilateral TPJ and MPPC in men compared to women during both self- and other-processing.

#### Small Evidence for Sex Differences

Here we showed that women and men had higher gender-congruent activation in bilateral amygdala across all conditions and specific to the self-condition in the right putamen. The putamen forms part of the basal ganglia that is involved in movement and reward processing by means of dopaminergic signaling (Schultz, 2016). This finding could point to a greater reward value of same sex attributes in women and men but this is limited due to the lack of correlations between neural activations and behavior that could help to inform the meaning of brain activation. In a similar vein comes the gender-congruent activation in bilateral amygdala. The amygdala is known for its involvement in the processing of emotional information (Lindquist et al., 2012; Dricu and Frühholz, 2016) and is also generally considered as a salience detector (Sander et al., 2003). Thus, another tentative interpretation for our results could be the increased salience of gender-congruent items in women and men leading to gender-congruent activation in the amygdala. Of further note, regressions of sex hormone levels on whole brain activation revealed stronger activation in left insula and left postcentral gyrus with rising progesterone and testosterone levels, respectively in women during self-processing of male attributes. None of these regions is located in the vicinity of the anterior cingulate cortex which was identified to be most strongly active during self compared to other processing in general (see “General Effects of Self- and Other-Appraisal”). Only the insula has been repeatedly implicated in self-referential processes (Enzi et al., 2009; Modinos et al., 2009) which could thus speak for a further pronunciation of self-related processes in association with progesterone levels. However, the separate correlations of sex hormone levels with the activations in several regions of interest yielded no conclusive pattern, implicating only higher bilateral amygdala activation with higher estradiol levels during presentation of male attributes in men. Human research still lacks a clear understanding of the cognitive effects of changes in sex hormone levels which has been most consistently investigated within women (e.g., Sundström-Poromaa and Gingnell, 2014; Toffoletto et al., 2014) however with no clear conclusions. In our experiment a multitude of statistical comparisons was performed as we analyzed women and men separately for female and male attributes in several regions. Therefore our data can only be considered preliminary and need further experimental support to corroborate and further specify them before strong conclusions can be derived.

### General Effects of Self- and Other-Appraisal

However, based on our study, we were able to give substantial evidence for a general neural difference between self- and other processing which we therefore want to explain in a bit more detail here.

#### The Self

Our results show that a cluster in the left anterior cingulate cortex extending to the insula was more strongly active during the self- compared to the other-conditions which is in line with previous reports showing stronger insular activation for self-processing compared to familiarity judgments (Qin et al., 2012). Especially the anterior insula has been repeatedly related to the awareness for internal body states (Craig, 2004, 2009) and was suggested to code emotional salience (Northoff et al., 2011). Thus, it does not come as a surprise that self-referential processing involves the anterior insula (Enzi et al., 2009; Modinos et al., 2009) suggesting that stronger personal involvement during self-reflection shares part of the neural substrates important for coding of emotional salience.

#### Others

Our results furthermore show that a cluster in the precuneus was more strongly activated during the other compared to the self-condition. The precuneus is classically involved in a variety of cognitive and emotional functions, such as mental and motor imagery (Cavanna and Trimble, 2006) but also social cognition, self-agency and self-activation (e.g., Vogeley and Fink, 2003). Interestingly, the precuneus is also an important part of the default mode network (Utevsky et al., 2014) but its activation seems to be more relevant for processing of other-related information. For example, stronger activation of the precuneus has been reported in participants deciding whether a sentence applied to another person or not (Veroude et al., 2013). Also, Qin et al. (2012) report that the precuneus preferentially responds to stimuli related to (personally) familiar people in contrast to self-specific stimuli. This fits with our findings as all participants were familiar with both actors and suggests that for ascribing the female or male traits to a prototypical woman (Julia Roberts) or man (Brad Pitt) their choices were based on classical gender stereotypes.

### Limitations

It has been shown that menstrual cycle phase influences attractiveness self-ratings of the own body (Durante et al., 2008) which might also translate to self-appraisals. For the current study we did not assess menstrual cycle phase or oral contraceptive intake and thus cannot rule out such hormonal influences played a confounding role (e.g., Pletzer et al., 2015). Further points of limitation refer to the small number of female and male participants potentially not allowing to detect more subtle sex differences. Furthermore, the number of different female and male items we used made it also impossible to balance each experimental condition for the same items. However, we point out that the mean ratings of female and male items did not differ between conditions and therefore this aspect is an unlikely confound in our experimental design. Finally, our participants were mainly students. To the present moment in Germany, there is still a divide between the number of female and male students in different fields of academia with 70–90% of male students in engineering subjects and around 80% of women in educational science. Both the subject of studies and the gender-ratio has been shown to impact stereotype processing, e.g., leading to a stronger stereotype threat when the gender-ratio is off-balanced (Murphy et al., 2007) or for students facing tasks that are off their subject of study (Sanchis-Segura et al., 2018). For this reason our study may not be able to allow general claims both within our sample of students and beyond academia. Other factors that may influence gender stereotyping are personality traits like the big five of personality research: openness to experience, conscientiousness, extraversion, agreeableness and neuroticism (Asendorpf, 2005). Unfortunately, we did not collect such information and await future studies to analyse how they might affect gender stereotyping.

## Conclusion

Measuring self- versus other-appraisals to explore behavioral and neural differences between healthy women and men revealed that both sexes self-ascribed more gender-congruent than -incongruent traits while also expressing a higher desire to exhibit more masculine traits. While fMRI did not detect general sex differences in the self- and other-conditions, some subtle differences were revealed between the sexes: both in right putamen and bilateral amygdala stronger gender-congruent activation was found which was however not associated with behavioral measures like the number of self-ascribed female or male attributes.

## Author Contributions

BD and UH contributed to conception and design of the study. JJ and KP collected the data. The majority of statistical analyses was performed by JH. ES contributed to statistical analyses. BD and JH wrote the first draft of the manuscript. All authors contributed remarks to improve the manuscript and approved the final version of the manuscript.

## Conflict of Interest Statement

The authors declare that the research was conducted in the absence of any commercial or financial relationships that could be construed as a potential conflict of interest.

## Footnotes

1 http://www.fil.ion.ucl.ac.uk/spm
