# Supplementary material for: Exploring Sex Differences in the Neural Correlates of Self-and Other-Referential Gender Stereotyping
Source: Front Behav Neurosci. 2019 Feb 18;13:31. doi: 10.3389/fnbeh.2019.00031 (PMC6387933; doi:10.3389/fnbeh.2019.00031)
Supplement: Supplementary file 1 [file Table_1.docx]

Supplementary Material

**Main effects of Condition.** Overall, main effects of Condition were detected in several ROIs. This means that in the precuneus processing of a prototypical man led to higher activation than the prototypical women (p = .049) and the self-condition (p = .016). In the left TPJ both the prototypical man (*p* < .001) and self (*p* < .001) led to higher activation compared to the prototypical woman. For the right TPJ higher activation during processing of the prototypical man compared to self (*p* < .001) and the prototypical woman (*p* < .001) were observed. The same was true for the MPPC with stronger activation in the prototypical man compared to self (*p* < .001) and the prototypical woman (*p* < .001). In the MPFC the activation was strongest for the self compared to the prototypical woman (*p* < .001) and man (*p* < .001) while the prototypical man led to higher activation compared to the prototypical woman (*p* < .001).

**Main effects of Attribute.** Female compared to male attributes caused higher activation in the left TPJ (*p* = .008), MPPC (*p* = .002) and MPFC (*p* = .007).
